# The problem with the ‘truth’: rethinking ground truth for artificial intelligence in endometriosis diagnosis

**DOI:** 10.1093/humrep/deag024

**Published:** 2026-02-25

**Authors:** Alison Deslandes, Yuan Zhang, Mathew Leonardi, Hsiang-Ting Chen, Gustavo Carneiro, Jodie Avery, George Condous, Steven Knox, M Louise Hull, the IMAGENDO Team, Alison Deslandes, Yuan Zhang, Mathew Leonardi, Hsiang-Ting Chen, Gustavo Carneiro, Jodie Avery, George Condous, Steven Knox, M Louise Hull

**Affiliations:** Robinson Research Institute, University of Adelaide, Adelaide, Australia; Specialist Imaging Partners, Adelaide, Australia; Robinson Research Institute, University of Adelaide, Adelaide, Australia; Australian Institute for Machine Learning, University of Adelaide, Adelaide, Australia; Robinson Research Institute, University of Adelaide, Adelaide, Australia; Department of Obstetrics and Gynecology, McMaster University, Hamilton, Canada; School of Computer and Mathematical Sciences, University of Adelaide, Adelaide, Australia; Centre for Vision, Speech and Signal Processing (CVSSP), University of Surrey, Guildford, United Kingdom; Robinson Research Institute, University of Adelaide, Adelaide, Australia; Robinson Research Institute, University of Adelaide, Adelaide, Australia; Endometriosis Ultrasound & Advanced Endosurgery Unit, Sydney Medical School Nepean, University of Sydney, Nepean Hospital, Sydney, Australia; Benson Radiology, Adelaide, Australia; Robinson Research Institute, University of Adelaide, Adelaide, Australia

**Keywords:** artificial intelligence, endometriosis, gynaecology, ultrasound, MRI, surgery, machine learning

## Abstract

Artificial intelligence (AI) is revolutionizing how we practice medicine. In areas where we have traditionally struggled, such as diagnosing endometriosis, AI has significant potential to improve the breadth and accuracy of diagnostic services offering a great benefit to patient care. When developing AI models for diagnosis, the ‘ground truth’ refers to the reference standard used in the labelling of the data used to train the model. Conventionally, in clinical medicine, we correlate any new diagnostic tool to the established ‘gold standard’, which in the case of endometriosis is laparoscopic visualization of lesions and histological confirmation. This method however is increasingly recognized as imperfect. Acknowledgement of the limitations of surgery and recent improvements in the diagnostic capability of imaging technologies to detect endometriosis, has created a situation where endometriosis no longer has one clear ‘gold standard’ for diagnosis. In this commentary, we will explore the impact of this on AI-driven endometriosis diagnostic tools and propose novel ways this could be addressed in the context of creating ground truths for endometriosis diagnosis.

## Introduction

Endometriosis is a common gynaecological condition believed to affect ∼190 million people globally (World Health Organisation, 2025). It is widely acknowledged that diagnosis of endometriosis is challenging due to varying symptoms, a lack of non-invasive testing which can identify all subtypes accurately, and a historical reliance on surgery to confirm diagnosis. Much effort has been expended in recent years to improve endometriosis diagnostics, particularly non-invasive options like biomarkers and imaging tests (Pascoal *et al.*, 2022). Given the numerous challenges in diagnosis, and the power of artificial intelligence (AI) in healthcare, it stands to reason that AI will have a role to play in diagnosis of endometriosis in the near future (Sivajohan *et al.*, 2022; Avery *et al.*, 2024b; Deslandes *et al.*, 2024).

Although surgical visualization of lesions has long been considered the ‘gold standard’, recent updates to clinical practice guidelines from international societies like ESHRE and the Royal Australian and New Zealand College of Obstetrics and Gynaecology (RANZCOG) no longer specify this to be the case (Becker *et al.*, 2022; Royal Australian and New Zealand College of Obstetrics and Gynaecology (RANZCOG) 2025). This change has largely been prompted by the improvement in imaging technologies and their diagnostic capabilities in recent years. It has however, created a situation where endometriosis no longer has one clear ‘gold standard’ for diagnosis (Pascoal *et al.*, 2022), a problem which raises the question, *what should constitute a ground truth in endometriosis diagnosis?* In this commentary, we hope to shine a spotlight on the challenges of defining ground truth in endometriosis diagnosis, drawing on insights from our own AI research. By sharing potential solutions, we aim to spark a global conversation and drive the innovation needed to push endometriosis diagnostics into a new digital era.

## What is a ground truth?

When developing AI models for diagnosis, the ‘ground truth’ refers to the reference standard use in the labelling of the data used to train the model (Drukker *et al.*, 2020; Willemink *et al.*, 2020). For instance, if training an AI model to diagnose endometriosis from MRI with a supervised learning approach, it would be reasonable to have an expert radiologist review and ‘label’ the images. These labelled images would then be used to teach the model how to read the MRI and potentially replicate the opinion of that expert radiologist in every instance. In clinical studies, best practice dictates that the accepted ‘gold standard’ is used as the reference standard to which other tests should be compared. Therefore, our historical use of surgery (±histology) as the reference standard would dictate that this should be our reference standard in endometriosis diagnosis from MRI images. However, our current lack of clinical clarity around what is the ‘gold standard’, and the known limitation of surgery and histology, means the choice of ground truth needs a considered approach when it comes to the creation of AI tools for the purpose of endometriosis diagnosis.

## The current potential ‘ground truths’ in endometriosis diagnosis

Currently, there are three main ‘human interpretation’ tools considered ‘diagnostic’ of endometriosis. These are imaging (through direct visualization of endometriosis lesions using ultrasound or MRI), surgery (through direct visualization of endometriosis lesions by the surgeons), and histology (through identification of pathological signs of endometriosis identified in surgical biopsies) (Becker *et al.*, 2022; Pascoal *et al.*, 2022). Each of these has strengths and limitations in their current form which impacts their utility as a ground truth for AI model training (Pascoal *et al.*, 2022).

### Limitations of imaging as ground truth

Imaging diagnosis of endometriosis has progressed rapidly in the past few decades and is now regarded as a highly accurate method of diagnosing ovarian endometriomas and deep endometriosis (Nisenblat *et al.*, 2016; Pascoal *et al.*, 2022; Avery *et al.*, 2024a). However, its ability to detect superficial endometriosis is limited, with poor sensitivities reported in literature (Bailey *et al.*, 2024; Freger and Leonardi, 2025; Mick *et al.*, 2025). As such, the widely accepted actuality is that negative imaging does not exclude endometriosis, and that there is a high false-negative rate (Guerriero *et al.*, 2016; Becker *et al.*, 2022; Deslandes *et al.*, 2024; Royal Australian and New Zealand College of Obstetrics and Gynaecology (RANZCOG) 2025).

In addition to this, imaging has several other limitations. Most notably, the accuracy of endometriosis imaging (both transvaginal ultrasound (TVUS) and MRI) is very dependent upon those performing or interpreting the imaging. Limited global availability of imaging experts with sufficient expertise to accurately diagnose endometriosis, compels scrutiny before the use of imaging reports as an AI ground truth is adopted, to ensure ‘labellers’ (i.e. clinicians reporting the imaging) have sufficient expertise in endometriosis detection (Avery *et al.*, 2026). Additionally, despite efforts through robust consensus publications aiming to standardize imaging protocols and reporting (Guerriero *et al.*, 2016; Bazot *et al.*, 2017; Jha *et al.*, 2020), there still exists significant variation in techniques and definitions of lesions across centres. This can cause potential for inconsistent or inaccurate labelling of data from different centres or geographic locations; a problem referred to as ‘noisy labels’ in the field of computer science.

Furthermore, current imaging modalities for endometriosis (TVUS and MRI) provide structural detail but cannot accurately assess the metabolic activity or the symptom-generating potential of lesions. Although TVUS may offer limited biofeedback through elicited tenderness and MRI can infer activity from blood products, neither can truly identify which lesions drive symptoms. Emerging nuclear medicine techniques may eventually offer this metabolic insight with a similarly non-invasive nature to TVUS and MRI (University of Oxford, 2024).

Imaging as a ground truth holds a key strength. It is feasible to perform imaging on most people, even in the absence of symptoms, given its non-invasive nature in comparison to surgery. This accessibility opens the possibility of collecting more diverse datasets, a broader population of patients than is possible with surgical diagnostics. However, there is currently little practice of performing imaging on asymptomatic people whether in clinical screening programs or research settings.

### Limitations of surgical visualization as a ground truth

Surgical visualization of endometriosis lesions has historically been considered the ‘gold standard’ for diagnosis and still is regarded by many clinicians and patients as the most reliable test. From a clinical perspective, it would stand to reason that surgical outcome (±histology) should also be the default option for ground truth in AI development. Surgery undoubtedly carries the highest detection rate of overall endometriosis (when all three subtypes [superficial, ovarian, and deep endometriosis] are grouped), making it a strong option as a ground truth. However, several limitations question its status as a ‘ground truth’. Firstly, given the invasive nature of surgery, the high cost, and associated risks, surgery is used only in cases where there is a compelling clinical reason to perform the procedure. Thus, surgery as a gold standard has an inherent bias that only those very likely to have disease will undergo this diagnostic procedure, and those with a low pre-procedure probability (i.e. those deemed likely to have a negative finding) are often excluded (Koninckx *et al.*, 2021; Pascoal *et al.*, 2022; Harder *et al.*, 2024). Additionally, through the integration of pre-surgical imaging assessments, we may be starting to witness surgical datasets that disproportionally represent those with advanced endometriosis identified on imaging. This introduces a bias to resultant AI models, limiting the generalizability of any algorithm to those with milder disease. On the other hand, patients that suffer from persistent pelvic pain, with frequent presentations to their gynaecologist may also have a higher surgical intervention rate due to the impact of their symptoms, regardless of their ultimate disease presence/absence. Thus, using surgery as a ground truth holds potential to introduce bias into any AI model; a complication which is to be avoided in model development wherever possible.

Surgical diagnosis/confirmation of endometriosis is not infallible. When therapeutic surgery is distinguished from diagnostic surgery, the ability for surgery to be a good diagnostic test for endometriomas and deep endometriosis plummets, as surgeons cannot see through the outer capsule of an ovary containing a cyst and deep endometriosis is often buried under adhesions and/or within the muscular layer of visceral organs (Goncalves *et al.*, 2021). When it comes to superficial endometriosis, some lesions, particularly clear lesions, may be occult and not visible during surgery (Hsu *et al.*, 2010; Khan *et al.*, 2014; Deslandes *et al.*, 2024). Indeed, it has even been shown that endometriosis can be identified histologically when the peritoneum appears normal (Gubbels *et al.*, 2020). Like imaging diagnostics, the experience and intent of the surgeon is critical, with less experienced surgeons more likely to overlook endometriosis or elect not to excise and/or biopsy lesions in close proximity to structures such as bowel or ureter. Together, these factors can result in false-negative findings at surgery (Pascoal *et al.*, 2022). In the clinical context of fertility-sparing surgery, less radical surgical approaches are often taken, intentionally leaving behind endometriosis lesions. Bowel endometriosis, for example, may be retained by the patient if removal is felt to be unhelpful to a fertility journey or too risky for the patient when they are making informed decision about their care. In these cases, there is no possible surgical or histologic confirmation, relying solely on imaging findings. Conversely, a false-positive surgical finding is possible. The surgeon’s desire to help the patient achieve diagnostic clarity and therapeutic benefit typically carries a broad expectation that surgeons will biopsy/remove anything that looks remotely abnormal, raising potential for surgical false positives.

Like imaging, though perhaps to an even greater extent, there is considerable variation in how surgeons document and report findings (Koninckx, 1998; Vermeulen *et al.*, 2021). This has certainly been our experience of attempting to collect ‘real-world’ surgical data from various centres, with extensive inconsistency in descriptions of technique, findings, and staging. While several established reporting systems for surgical endometriosis exist, there is inconsistency in their clinic uptake (Becker *et al.*, 2014; Keckstein *et al.*, 2021; Abrao *et al.*, 2023). This variability increases the risk of inconsistent data labelling across different centres.

### Limitations of histology as a ground truth

The use of histology as a gold standard for endometriosis diagnosis is also problematic. Despite excision surgery being widely considered the optimal surgical approach, ablation surgery is often still commonly used for multiple reasons (Pundir *et al.*, 2017; Zanelotti and DeCherney, 2017; Bignardi *et al.*, 2019). The use of ablation surgery means that tissue cannot be sent for histological diagnosis as it will be destroyed. Furthermore, even with excision of lesions, full evaluation of the lesion histologically can be difficult due to small/insufficient tissue sample size, destruction of lesions during the excision process (e.g. burn or crush artifacts), or processing errors (Clement, 2007; Pascoal *et al.*, 2022). In research, lesions must be serial sectioned all the way through and sections stained at intervals to identify smaller lesions, a process too time consuming for routine pathological evaluation. The expertise of the technologist in processing tissues, and the pathologist in identifying sometimes subtle signs of endometriosis is critical. Even then, some classic endometriosis lesions (e.g. fibrotic lesions) may not meet histological diagnostic criteria. Lastly, not all lesions will be sent for histology as this is not always clinically necessary or feasible (Pascoal *et al.*, 2022).

## The problem of noisy labelling

A discussion of endometriosis diagnostics and ground truths would not be complete without simultaneously considering the challenge of noisy labelling. In the development of AI models, inconsistent or inaccurate labels create noise and bias in training data, a complication which is to be avoided as much as possible (Karimi *et al.*, 2020). In other words, if the ‘truth’ you are training your AI on, whether it be expertly labelled imaging or surgical/histology findings, is incomplete, inconsistent, or just plain wrong, the AI will learn to match those mistakes (Drukker *et al.*, 2020) and ultimately overfit the training data which will generalize poorly to previously unseen testing data. In the real world, however, there will always be some degree of difference in human interpretation, whether the modality be imaging, surgery, or histology (Deslandes *et al.*, 2025a), so dealing with ‘noisy labels’ is a common consideration in the field of machine learning (ML) and AI. Thus, in addition to strategies for refining the ground truth for endometriosis, consensus approaches to dealing with labelling noise are essential (Plank, 2022; Deslandes *et al.*, 2025a), and strategies to manage this are critical in the development of endometriosis diagnostics.

## Potential solutions to overcome current limitations

Given each diagnostic approach has strengths and limitations, all hold potential to be used as a ground truth. Innovative solutions to overcome the limitations may present the solution. Importantly, selection bias represents a fundamental structural limitation across all currently available ground truth modalities in endometriosis research. Because endometriosis diagnosis follows a symptom-driven investigative pathway, imaging datasets are enriched for symptomatic individuals, and surgical datasets are further enriched for high-suspicion cases, leaving asymptomatic, early-stage, or atypical disease systematically under-represented. Consequently, AI models trained on existing data are likely to learn patterns of ‘investigation-worthy’ disease, with uncertain performance in the very cases where earlier detection may be most valuable. Innovative solutions to overcome the limitations may present the solution. This not only requires technological innovation however, but a shift in our traditional clinical and research thinking towards diagnostic accuracies and ‘gold standards’ (Pascoal *et al.*, 2022). Here, we present several options we believe could be feasible and warrant consideration at a professional level. Whichever methods are eventually utilized going forward, it is imperative that their impact on any resultant algorithm trained with that ground truth is appreciated by the clinicians ultimately using them (Drukker *et al.*, 2020; Alowais *et al.*, 2023).

### Ground truth from a single modality

In a single modality ground truth approach, the relevant modality (e.g. imaging or surgery) would be used only as a ground truth to that modality. For example, the AI model is trained to diagnose endometriosis from an ultrasound with labels produced by an expert sonographer as they interpreted the ultrasound. This approach may be very well suited to application of AI diagnosis like developing image interpretation algorithms, where the labels of an expert in that modality can be used as the ground truth. The simplicity of this approach makes understanding the outputs for the clinicians using these tools relatively straightforward. Additionally, a single modality approach could theoretically optimize the diagnostic capability of that model for its specific intention and remove the risk of inherited biases or limitations from another modality (such as using a surgical ground truth for imaging models). This does however limit the ability to draw upon the strength of other modalities which could potentially optimize model performance if used correctly (Zhang *et al.*, 2023). Furthermore, the tool will only be as good as the label used to build it. Although AI models can incorporate strategies to correct for labelling inconsistencies, the accuracy of any system ultimately remains constrained by the quality of the labels it is trained on. The tool can only be as reliable as its underlying annotations; therefore, any labelling errors have the potential to be propagated into the model itself, contributing to an inaccurate ground truth and limiting overall performance. As such, if the labeller makes an error, so will the tool, potentially creating an inaccurate ground truth.

### Ground truth from multiple modalities

Rather than being reliant on one ‘gold standard’ as has traditionally been the case in clinical research, using a multiple modality consensus approach to creating a ground truth for AI models aiming to diagnose endometriosis may be a solution (Yu *et al.*, 2020). Such an approach would combine multiple data points (e.g. MRI findings, TVUS findings, surgical findings, histological features, and clinical presentation [e.g. pain, infertility]) into a composite label. This approach replicates the work of human experts performing diagnosis more closely, as humans usually consider multiple variables (clinical symptoms, personal and family history, imaging, etc.) to form a diagnosis best matched to their expert opinion. AI models however lack the ability to reason like humans and do not appreciate that each presentation is nuanced (Drukker *et al.*, 2020). This approach would almost certainly result in very ‘noisy’ labels. However, in the context of AI tool creation, more information is better and noise can be dampened using AI approaches. Solutions to deal with labelling noise include majority voting or weighted consensus methods (Petashvili *et al.*, 2024). One such strategy specific to endometriosis diagnostics has been developed by Wang *et al.* (2024) called Human-AI Collaborative Multi-Modal Multi-Reader learning for endometriosis diagnosis (HAICOMM), which works to manage labelling inconsistencies in endometriosis imaging by combining multi-rater learning (to refine inconsistent/noisy clinician labels), multi-modal learning (to utilize T1/T2 MRI data), and human-AI collaboration.

A multi-modality ground truth approach requires access to wide datasets which development teams may not have. One such approach to overcome this limitation and leverage the value of multi-modal algorithms without access to paired datasets was proposed by Zhang *et al.* (2025). By training a multi-modal classifier on unpaired TVUS and MRI data, a model was created that could transfer knowledge across modalities, improving MRI-based classification of endometriosis signs while preserving the high accuracy of TVUS.

A multi-modal approach also allows the approach used by Zhang *et al.* (2025) for implementation of AI techniques which can help to mitigate against the issue of noisy labels and ground truthing imperfections which will likely be unavoidable in endometriosis diagnostics. Techniques such as semi-supervised learning can be utilized to combine a small, well-labelled dataset from one modality (such as surgery) with larger unlabelled data (such as MRI) to generalize the findings of the smaller dataset to a broader population.

### Use of longitudinal outcomes as proxies to a ground truth

One area in which ML is revolutionizing medicine is in predictive medicine (Phillips *et al.*, 2022; Dixon *et al.*, 2024; Khalifa and Albadawy, 2024). By analysing data from a diverse range of sources, such as electronic health records and patient registries, then combining with ML, it is possible to create avenues that enhance disease prediction and personalize management (Alhumaidi *et al.*, 2025). In the context of a ground truth for endometriosis diagnosis, a longitudinal outcomes approach would assess follow-up of symptom improvement as validation of initial diagnosis, rather than assessment of the initial ‘findings’. Given the challenges associated with endometriosis management, especially the lack of current curative treatment options, an ‘outcome’ based approach may be effective, especially as the diagnostic objective should be to facilitate appropriate and beneficial treatments (Franye *et al.*, 2023; Fallon *et al.*, 2024). Conversely, it may be impractical to use in the context of endometriosis, where it is well appreciated that symptoms do not match disease stage/severity. Endometriosis is an exceedingly complex whole-body condition, with a range of implications and outcomes, so the feasibility of this approach is difficult to determine. Outcome-based approaches have not previously been applied in human-led endometriosis studies. However, they may ultimately be more clinically beneficial than traditional interpretation-focused methods used in clinical, imaging, or pathological diagnosis. It must be noted however that ethical deployment of any AI system, but especially one designed and modelled on a novel ground truth solution (or particularly noisy labelling) requires transparency around uncertainty, the measured outputs, and validation on patient-centred outcomes. It is vital that any tool undergo a rigorous external validation process, and that the clinicians ultimately using these tools to assist in their clinical decision-making are aware of the limitations they may be working with. Clinicians must also be comfortable explaining these to their patients to ensure informed consent to use such tools is given.

### Standardized imaging and surgical protocols with AI-compatible labels

While innovative approaches to developing ground truths are essential, the need for development and adoption structured reporting tools for routine clinical use cannot be overlooked. Methods such as the World Endometriosis Research Foundation Endometriosis Phenome and Biobanking Harmonisation Project (WERF EPHect) tools, have been developed to standardize reporting and create robust research databanks across centres. However, their use in daily clinical practice is limited at best. The WERF EPHect tools cover a range of endometriosis-related reporting (e.g. surgery, pain) however, it is imperative that moving forward, such tools map well to the development of AI models. This needs to be system-wide in imaging, surgery, and histology, as well as a consideration for any future diagnostic tools which may become mainstream like biomarkers (Fassbender *et al.*, 2012; Bilibio *et al.*, 2014; Acimovic *et al.*, 2016; Joshi *et al.*, 2017; Ferrier *et al.*, 2023; Hon *et al.*, 2023; Schoeman *et al.*, 2025). While standardization of data will not solve all the problems, it will remove some of the current limitations which exists and undoubtably result in higher quality data for AI development, which will in turn lead to better performing models.

### Ground truth as a likelihood or uncertainty-aware label

While in medicine we feel most comfortable with diagnostic certainty and ‘confirmed’ diagnosis, in practice, the process of forming a diagnosis of endometriosis is rarely binary—especially given the limitations of our established ‘gold standards’. Imaging findings, surgical reports, and histology often diverge, and even experts will frequently describe levels of suspicion rather than absolute presence or absence. Some discrepancies reflect inter-observer variation, others reflect inherent differences between modalities (e.g. ultrasound vs MRI), and some reflect the biological complexity of the disease itself. Forcing such complex and sometimes conflicting information into a single ‘yes/no’ label risks oversimplification and reduces the clinical credibility of any AI system trained on it. An uncertainty aware approach would rather present a likelihood of endometriosis being present rather than an absolute yes/no result. Diagnostic uncertainties could be directly encoded into the ground truth. Rather than treating differences between modalities or experts as ‘noise’ to be removed, they can be represented as probabilistic or ‘soft’ labels that reflect diagnostic confidence. For example, if three radiologists interpret an ultrasound sign differently, the label could reflect the distribution of their opinions rather than a forced consensus (Wang *et al.*, 2024; Deslandes *et al.*, 2025b). Similarly, discrepancies between various modalities could be encoded as weighted likelihoods instead of discarded as inconsistency. This approach recognizes that uncertainty is an inherent feature of endometriosis diagnosis, not simply an error.

Training AI models on such uncertainty-aware ground truths would allow the system to learn not only the likely diagnosis, but also the degree of confidence associated with it. This mirrors clinical practice more closely, where clinicians integrate likelihoods and confidence levels into their decision-making. Importantly, AI outputs that include confidence measures could also highlight cases where additional imaging or surgical confirmation may be warranted. In this way, encoding uncertainty makes AI systems more transparent, safer for clinical use, and better aligned with the way clinicians already think and work.

In practice, adopting a likelihood ground truth would require an operational and cultural shift in how we approach our clinical workflow, not simply a change in how we annotate data. If such an approach were to be used, we would need to use reporting systems that allow confidence scoring, which our current infrastructure and systems often do not allow for in the field of endometriosis diagnostics. However, this is an approach commonly accepted in the imaging assessment of ovarian lesions, where accurately distinguishing benign from malignant can be challenging. Well-established diagnostic systems such as IOTA-ADNEX and O-RADS encourage reporting on probability of malignance (i.e. almost certainly benign, low risk, intermediate risk, high risk) (Calster *et al.*, 2014; Strachowski *et al.*, 2023). These systems are universally accepted and have seen arguably good uptake into clinical practice to facilitate appropriate care and treatment. There is no reason similar systems could not be adopted for endometriosis to assist with prediction of not only the presence of endometriosis lesions, but also the likelihood of these being related to symptoms.

## Conclusion

Endometriosis is a complex condition which creates challenges not only in clinical care but in AI tool development. Given the current lack of a clear, perfect ‘gold standard’ it is essential to discuss what a suitable ground truth in AI for endometriosis diagnosis looks like. Increasingly, the size and quality of our datasets will iteratively improve the robustness of predictive algorithms and ultimately provide accuracy assessments of both imaging and surgical findings. Collaboration between clinicians, data scientists, and patients to ensure AI models are clinically meaningful, not just technically impressive will be essential. It is our opinion that addressing the challenges involved in developing accurate and predictive non-invasive diagnostic tools for endometriosis is most likely to integrate a multimodal approach. Already, inconsistencies between modalities are questioning our historical concept that surgical reporting is ‘gold standard’ when it, like imaging reporting, has always been subject to human bias. Although highly influenced by the quality of imputed data, this will rapidly improve over time and AI can provide an objective data-driven approach to diagnostics, likely to reduce diagnostic delay for 190 million people globally affected by endometriosis. We believe AI will be a revolution in endometriosis management, but ultimately the skills and clinical decision-making that only humans can bring, will still be needed in developing a clear pathway to a better quality of life.

## Authors’ roles

A.D.: Conceived and led the manuscript concept; undertook the primary literature review; drafted the initial manuscript; integrated feedback from all co-authors; coordinated revisions; and provided substantial critical intellectual input. Y.Z.: Contributed to manuscript conception and design, particularly regarding AI methodology; provided technical input on preliminary data interpretation; critically revised the manuscript for important intellectual content; and approved the final version. M.L.: Contributed to conception and clinical context; provided specialist input on gynaecological ultrasound, surgery and endometriosis; reviewed and revised the manuscript critically for clinical accuracy; and approved the final version. H.-T.C.: Provided technical expertise in AI design and implementation; assisted in interpretation of preliminary study data; revised the manuscript critically for technical accuracy; and approved the final version. G.C.: Contributed to AI technical aspects; critically revised the manuscript for intellectual content; and approved the final version. J.A.: Contributed to study design and interpretation; provided preliminary data analysis and evidence synthesis; revised the manuscript critically; and approved the final version. G.C.: Provided clinical expertise in endometriosis, surgery and ultrasound; contributed to study design and interpretation; critically revised the manuscript; and approved the final version. S.K.: Provided clinical expertise in endometriosis and MRI; revised the manuscript critically for accuracy; and approved the final version. M.L.H.: Provided expertise in endometriosis, reproductive medicine and research design; contributed to the conception and interpretation of findings; critically revised the manuscript for intellectual content; and approved the final version. All authors meet the four ICMJE authorship criteria: (i) substantial contributions to conception/design/data; (ii) drafting/revising; (iii) final approval; and (iv) accountability for accuracy and integrity.
